# Palmitoylethanolamide and luteolin ameliorate development of arthritis caused by injection of collagen type II in mice

**DOI:** 10.1186/ar4382

**Published:** 2013-11-18

**Authors:** Daniela Impellizzeri, Emanuela Esposito, Rosanna Di Paola, Akbar Ahmad, Michela Campolo, Angelo Peli, Valeria Maria Morittu, Domenico Britti, Salvatore Cuzzocrea

**Affiliations:** 1 Department of Biological and Environmental Sciences, University of Messina, Messina, Italy; 2 Clinical Veterinary Department Alma Mater Studiorum, University of Bologna, Bologna, Italy; 3 Department of Health Sciences V. le Europa, Campus S. Venuta, Germaneto, 88100 Catanzaro, Italy; 4 Manchester Biomedical Research Centre, Manchester Royal Infirmary, University of Manchester, Manchester, UK

## Abstract

**Introduction:**

*N*-palmitoylethanolamine (PEA) is an endogenous fatty acid amide belonging to the family of the *N*-acylethanolamines (NAEs). Recently, several studies demonstrated that PEA is an important analgesic, antiinflammatory, and neuroprotective mediator. The aim of this study was to investigate the effect of co-ultramicronized PEA + luteolin formulation on the modulation of the inflammatory response in mice subjected to collagen-induced arthritis (CIA).

**Methods:**

CIA was induced by an intradermally injection of 100 μl of the emulsion (containing 100 μg of bovine type II collagen (CII)) and complete Freund adjuvant (CFA) at the base of the tail. On day 21, a second injection of CII in CFA was administered. Mice subjected to CIA were administered PEA (10 mg/kg 10% ethanol, intraperitoneally (i.p.)) or co-ultramicronized PEA + luteolin (1 mg/kg, i.p.) every 24 hours, starting from day 25 to 35.

**Results:**

Mice developed erosive hind-paw arthritis when immunized with CII in CFA. Macroscopic clinical evidence of CIA first appeared as periarticular erythema and edema in the hindpaws. The incidence of CIA was 100% by day 28 in the CII-challenged mice, and the severity of CIA progressed over a 35-day period with a resorption of bone. The histopathology of CIA included erosion of the cartilage at the joint. Treatment with PEA or PEA + luteolin ameliorated the clinical signs at days 26 to 35 and improved histologic status in the joint and paw. The degree of oxidative and nitrosative damage was significantly reduced in PEA + luteolin-treated mice, as indicated by nitrotyrosine and malondialdehyde (MDA) levels. Plasma levels of the proinflammatory cytokines and chemokines were significantly reduced by PEA + luteolin treatment.

**Conclusions:**

We demonstrated that PEA co-ultramicronized with luteolin exerts an antiinflammatory effect during chronic inflammation and ameliorates CIA.

## Introduction

Rheumatoid arthritis (RA) is an autoimmune disease that results in multiple joint inflammations with subsequent destruction of joint cartilage and erosion of bone. Type II collagen-induced arthritis (CIA) in the mouse is a useful model of RA, as it possesses many of the cell and humoral immunity characteristics found in human RA [1]. The pathogenesis of CIA is dependent on the host’s response to type II collagen challenge and the subsequent generation of antibodies that recognize collagen-rich joint tissue [1]. Moreover, the recruitment and activation of neutrophils, macrophages, and lymphocytes into joint tissues and the formation of the pannus are hallmarks of the pathogenesis of both CIA and human RA. Recently, it was demonstrated that interleukin (IL)-8, MIP-lα, MIP-1β, and RANTES are differentially chemotactic for lymphocyte subsets [2]. The current treatments for delaying RA progression include several disease-modifying antirheumatic drugs (DMARDs) and biologic agents that act as immunomodulatory drugs in RA [3], some also act by inhibiting cytokines and endothelial cell proliferation [4]. Moreover, all of these compounds have potentially serious side effects; substantial differences in toxicity occur among DMARDs [5].

*N*-palmitoylethanolamine (PEA) is an endogenous fatty acid amide belonging to the family of the *N*-acylethanolamines (NAEs). PEA is an important analgesic, antiinflammatory, and neuroprotective mediator, acting at several molecular targets in both central and sensory nervous systems as well as in immune cells [6]. Several mechanisms have been proposed to explain the antiinflammatory and antihyperalgesic effects of PEA, including: (a) the activation of a cell-surface receptor (that is, the “CBn” (or CB2-like) or, alternatively, the orphan GPR55 receptor) or otherwise a nuclear receptor of the peroxisome proliferator-activated receptors (PPARs) family [7]; (b) the downmodulation of mast cell hyperactivity (ALIA mechanism) [8]; (c) an action as “entourage” compound (that is, the augmentation of eCBs activities at their receptors and/or the inhibition of eCBs degradation [9]). Although its presence in mammalian tissues has been known since the 1960s, PEA has emerged only recently among other bioactive *N*-acylethanolamines as an important endogenous lipid modulator that, because of its chemical stability, can be also administered exogenously as the active principle of current antiinflammatory and analgesic preparations [10]. Moreover, some evidence indicates that superoxide anions (O_2_^-^) perpetuate the chronic inflammatory state associated with RA. Thus, it follows that one therapeutic approach to treat RA is to remove these reactive oxygen species (ROSs). Osteoclasts, chondrocytes, synovial cells neutrophils/macrophages, and fragmented particles of degraded extracellular matrix are excellent sources of superoxide [11], as suggested from studies performed in animal models of arthritis [12], as well as in pilot experiments carried out in patients with active RA [13].

Flavonoids are natural products widely distributed in the plant kingdom and currently consumed in large amounts in the daily diet. Dietary flavonoids possess multiple neuroprotective actions in central nervous pathophysiologic conditions, including depression [14], and it was reported that naringenin has potent antidepressant-like properties through central serotonergic and noradrenergic systems. It was further suggested that dietary flavonoids possess a therapeutic potential in disorders especially in which the monoaminergic system is involved [14]. Luteolin is a common flavonoid found in many types of plants, such as *Apium graveolens* L. var. dulce [15], *Petroselium crispum*[16], and *Capsicum annuum* L. var. grossum [17]. It has various pharmacologic activities, such as antioxidant and anticancer action [18].

The purpose of study was to determine whether prolonged administration of co-ultramicronized PEA + luteolin (PEA-LUT) would ameliorate development of arthritis by using a CIA model.

## Methods

### Animals

Male DBA/1J mice (9 weeks; Harlan Nossan, Italy) were used for these studies. Mice were housed in individual cages (two for each group) and maintained under a 12:12 light–dark cycle at 21°C ± 1°C and 50% ± 5% humidity. The animals were acclimated to their environment for 1 week and had *ad libitum* access to tap water and standard rodent diet. All animal experiments complied with regulations in Italy (D.M. 116192), Europe (O.J. of E.C. L 358/1 12/18/1986), and the United States (Animal Welfare Assurance Number A5594-01, Department of Health and Human Services, USA). All behavioral testing was conducted in compliance with the NIH laboratory animal care guidelines and with protocols approved by the Institutional Animal Care and Use Committee (Council directive 87–848, October 19, 1987, Ministère de l’Agriculture et de la Forêt, Service Vétérinaire de la Santé et de la Protection Animale, permission 92–256 to SC). The study was approved by the University of Messina Review Board for the care of animals (PRIN ID 1042).

### Experimental groups

Mice were divided into the following five experimental groups:

**CIA-Control:** mice were subjected to collagen-induced arthritis and administered 200 μl of 10% ethanol solution (i.p., vehicle for PEA) every 24 hours, starting from day 25 to day 35 (*n* = 20).

**CIA + LUT:** mice subjected to collagen-induced arthritis were administered LUT (1 mg/kg, 10% ethanol, i.p.) every 24 hours, from day 25 to day 35 (*n* = 20).

**CIA-PEA:** mice subjected to collagen-induced arthritis were administered PEA (10 mg/kg, 10% ethanol, *i.p*.) every 24 hours, from day 25 to day 35 (*n* = 20).

**CIA-PEA-LUT;** mice subjected to collagen-induced arthritis were administered PEA and luteolin (single treatment combination) (1 mg/kg, i.p.) every 24 h, starting from day 25 to day 35 (n = 20).

**Sham-Control:** mice subjected to an intradermal injection at the base of the tail of 100 μl of 0.01 *M* acetic acid, instead of the emulsion containing 100 μg of CII, were treated with 200 μl of 10% ethanol solution (i.p., vehicle for PEA), every 24 hours from day 25 to day 35 (*n* = 20).

**Sham-LUT:** mice subjected to an intradermal injection at the base of the tail of 100 μl of 0.01 *M* acetic acid instead of the emulsion containing 100 μg of CII, were administered LUT (1 mg/kg, 10% ethanol, i.p.), every 24 hours from day 25 to day 35 (*n* = 20).

**Sham-PEA:** mice subjected to an intradermal injection at the base of the tail of 100 μl of 0.01 *M* acetic acid instead of the emulsion containing 100 μg of CII, were administered PEA (10 mg/kg, 10% ethanol, i.p.), every 24 hours from day 25 to day 35 (*n* = 20).

**Sham-PEA-LUT:** mice subjected to an intradermal injection at the base of the tail of 100 μl of 0.01 *M* acetic acid instead of the emulsion containing 100 μg of CII, were administered PEA and luteolin (single-treatment combination) (1 mg/kg, i.p.), every 24 hours from day 25 to day 35 (*n* = 20).

PEA-LUT preparation was formulated through a co-ultramicronization process by jet milling technology. The ratio between PEA and luteolin is 10:1 by mass. The doses of PEA and LUT were chosen based on our recent studies [19,20] to compare possible differences with PEA-LUT formulation.

### Induction of CIA

The induction of CIA was performed as described in our previous study [21]. Chicken type II collagen (CII) was dissolved in 0.01 *M* acetic acid at a concentration of 2 mg/ml by stirring overnight at 4°C. Dissolved CII was frozen at -70°C until use. Complete Freund adjuvant (CFA) was prepared by the addition of *Mycobacterium tuberculosis* H37Ra at a concentration of 2 mg/ml. Before injection, CII was emulsified with an equal volume of CFA. On day 1, mice were injected intradermally at the base of the tail with 100 μl of the emulsion (containing 100 μg of CII). On day 21, a second injection of CII in CFA was administered.

### Clinical assessment of CIA

The development of arthritis in mice in all experimental groups was evaluated daily starting from day 20 after the first intradermal injection by using a macroscopic scoring system: 0 = no signs of arthritis; 1 = swelling and/or redness of the paw or one digit; 2 = two joints involved; 3 = more than two joints involved; and 4 = severe arthritis of the entire paw and digits [22]. Arthritic index for each mouse was calculated by adding the four scores of individual paws. Clinical severity was also determined by quantitating the change in the paw volume by using plethysmometry (model 7140; Ugo Basile).

### Behavioral assays

#### Rotarod

Locomotor abilities were assessed with a protocol previously used [23]. DBA/1J mice were given 3 days of training on the rotarod before disease induction. Trials were conducted, starting on day 20 after CIA induction, every 5 day until day 35. DBA/1J CIA mice treated with vehicle, or PEA (10 mg/kg, i.p.), or PEA-LUT (1 mg/kg, i.p.) were placed for 3 minutes on the rotating beam of a rotarod (Ugo Basile) that was rotating at a fixed rate of 16 rpm. Each mouse was given three trials, after which the average time that a mouse remained on the rotating beam was calculated.

#### Pain-sensitivity testing

Hotplate testing was used to evaluate pain sensitivity as previously described [24]. In brief, mice were placed on a 55°C hotplate and observed by two individuals masked to treatment. The latency to a behavioral response was recorded. Behaviors included rearing, paw licking, paw stamping, or jumping. Mice were removed from the hotplate after 30 seconds if no response was observed.

#### Thermal hyperalgesia

Hyperalgesic responses to heat were determined by the Hargreaves Method by using a Basile Plantar Test (Ugo Basile, Comeria, Italy) [25] with a cut-off latency of 20 seconds used to prevent tissue damage. Animals were allowed to acclimate within a Plexiglas enclosure on a clear glass plate in a quiet testing room. A mobile infrared generator was positioned to deliver a thermal stimulus directly to an individual hindpaw from beneath the chamber. The withdrawal latency period of inflamed paws was determined with an electronic-clock circuit and thermocouple. Foot-withdrawal latencies were taken on day 0 before CIA induction (baseline) and subsequently on days 25, 30, and 35 of the experimental period to determine the analgesic effect of PEA or PEA-LUT treatments. A significant (*P* < 0.05) reduction in paw-withdrawal latency over time is characterized as thermal hyperalgesia. Data obtained were converted to percentage maximal possible antinociceptive effect (%MPE) as follows: (response latency - baseline latency)/(cut-off latency - baseline latency) × 100.

### Histologic examination

On day 35, animals were killed while they were under anesthesia (sodium pentobarbital, 45 mg/kg, i.p), and paws and knees were removed and fixed in 10% formalin. The paws were then trimmed, placed in decalcifying solution for 24 hours, embedded in paraffin, sectioned at 5 μm, stained with hematoxylin/eosin, and studied by using light microscopy (Dialux 22 Leitz). The following morphologic criteria were considered: 0 = no damage; 1 = edema; 2 = inflammatory cell presence; and 3 = bone resorption.

### Staining of mast cells

Identification of mast cells was performed as described in previous studies [26]. Paw sections were cut 5 μm thick and stained with 0.25% toluidine blue, pH 2.5, for 45 minutes at room temperature. The sections were then dehydrated and mounted in xylene-based medium for viewing. Three nonsequential sections were chosen from one random block from each spinal cord for examination. All sections were evaluated at 200×, whereas some sections were photographed at 400× by using a Nikon inverted microscope.

### Immunohistochemical localization of chymase, tryptase, and nitrotyrosine

Immunohistologic analysis was performed as described in previous studies [26]. On day 35, the joints were trimmed, placed in decalcifying solution for 24 hours, and 8-μm sections were prepared from paraffin-embedded tissues. After deparaffinization, endogenous peroxidase was quenched with 0.3% H_2_O_2_ in 60% methanol for 30 minutes. The sections were permeabilized with 0.1% Triton X-100 in PBS for 20 minutes. Nonspecific adsorption was minimized by incubating the section in 2% normal goat serum in phosphate-buffered saline for 20 minutes. Endogenous biotin or avidin binding sites were blocked by sequential incubation for 15 minutes with avidin and biotin. Sections were incubated overnight with (a) anti-chymase antibody (1:100 in PBS, vol/vol) (DBA, Milan, Italy), (b) anti-tryptase antibody (1:500 in PBS, vol/vol), or (c) anti-nitrotyrosine rabbit polyclonal antibody (1:1,000 in PBS, vol/vol). Controls included buffer alone or nonspecific purified rabbit IgG. Sections were washed with PBS and incubated with secondary antibody. Specific labeling was detected with a biotin- conjugated goat anti-rabbit IgG and avidin-biotin peroxidase complex (Vector). To verify the binding specificity for chymase and tryptase, some sections were also incubated with only the primary antibody (no secondary) or with only the secondary antibody (no primary). To confirm that the immunoreaction for the nitrotyrosine was specific, some sections were also incubated with the primary antibody (anti-nitrotyrosine) in the presence of excess nitrotyrosine (10 m*M*) to verify the binding specificity. In these situations, no positive staining was found in the sections, indicating that the immunoreaction was positive in all the experiments carried out. Immunocytochemistry photographs (*n* = 5) were assessed with densitometry by using Optilab Graftek software on a Macintosh personal computer [27].

### Radiography

Radiography was performed as previously described [11]. The mice were anesthetized with sodium pentobarbital (45 mg/kg, i.p.). Mice were placed on a radiographic box at a distance of 50 cm from the x-ray source. Radiographic analysis (Philips X12, Germany) of normal and arthritic mice hindpaws was performed with a 40-kW exposure for 0.01 seconds. An investigator blinded to the treatment regimen scored the radiographs. The following radiographic criteria from hindlimbs were considered: score 0, no bone damage; score 1, tissue swelling and edema; score 2, joint erosion; and 3, bone erosion and osteophyte formation.

### Measurement of cytokines

Tumor necrosis factor-α (TNF-α), interleukin (IL)-6, and IL-1β levels were evaluated in the plasma from CIA and sham mice, as previously described [28]. The assay was carried out by using a colorimetric commercial ELISA kit (Calbiochem-Novabiochem Corporation, Milan, Italy) with a lower detection limit of 10 pg/ml.

### Measurement of chemokines

Levels of chemokines MIP-1α and MIP-2 were measured in the aqueous joint extracts. In brief, joint tissues were prepared by first removing the skin and separating the limb below the ankle joint. Joint tissues were homogenized on ice in 3-ml lysis buffer (PBS containing 2 m*M* PMSF, and 0.1 mg/ml (final concentration), each of aprotinin, antipain, leupeptin, and pepstatin A) by using Polytron (Brinkinarm Instruments, Westbury, NY, USA). The homogenized tissues were then centrifuged at 2,000 *g* for 10 minutes. Supernatants were sterilized with a millipore filter (0.2 μm) and stored at -80°C until analyzed. The extracts usually contained 0.2 to 1.5 mg protein/ml, as measured with a protein-assay kit (Pierce Chemical Co., Rockford, IL, USA). The levels of MIP-1α and MIP-2 were quantified by using a modification of a double-ligand method, as previously described [29]. In brief, flat-bottomed 96-well microtiter plates were coated with 50 μl/well of rabbit anti-cytokine antibodies (1 μg/ml in 0.6 *M* NaCl, 0.26 *m* H_3_BO_4_, and 0.08N NaOH, pH 9.6) for 16 hours at 4°C, and then washed with PBS, pH 7.5, 0.05% Tween 20 (wash buffer). Nonspecific binding sites on microtiter plates were blocked with 2% BSA in PBS and incubated for 90 minutes at 37°C. Plates were rinsed 4 times with wash buffer, and diluted aqueous joint samples (50 μl) were added, followed by incubation for 1 hour at 37°C. After washing of plates, chromogen substrate was added. The plates were incubated at room temperature to the desired extinction, after which the reaction was terminated with 50 μl/well of 3 *M* H_2_SO_4_ solution. The plates were then read at 490 nm in an ELISA reader. This ELISA method consistently had a sensitivity limit of about 30 pg/ml.

### Thiobarbituric acid-reactant substances measurement (MDA levels)

Thiobarbituric acid-reactant substances measurement, which is considered a good indicator of lipid peroxidation, was determined, as previously described [30]. Thiobarbituric acid-reactant substances were calculated by comparison with OD650 of standard solutions of 1,1,3,3-tetramethoxypropan 99% malondialdehyde bis (dymethyl acetal) 99% (MDA) (Sigma). The absorbance of the supernatant was measured with spectrophotometry at 650 nm.

### Myeloperoxidase (MPO) assay

Neutrophil infiltration into the inflamed joints was indirectly determinate by using an MPO assay, as previously described for neutrophil elicitation [31]. Tissue was prepared as described earlier and placed in a 50 m*M* phosphate buffer (pH 6.0) with 5% hexadecyltrimethyl ammonium bromide (Sigma Chemical). Joint tissues were homogenized, sonicated, and centrifuged at 12,000 *g* for 15 minutes at 4°C. Supernatants were assayed for MPO activity by using a spectrophotometric reaction with *O*-dianisidine hydrochloride (Sigma Chemical) at 460 nm.

### Materials

Unless otherwise stated, other compounds were obtained from Sigma-Aldrich Company (Milan, Italy). All chemicals were of the highest commercial grade available. All stock solutions were prepared in nonpyrogenic saline (0.9% NaCl; Baxter Healthcare Ltd., Thetford, Norfolk, UK) or 10% ethanol (Sigma-Aldrich).

### Data analysis

All values in the figures and text are expressed as mean ± standard error (SEM) of the mean of *n* observations. For the *in vivo* studies, *n* represents the number of animals studied. In the experiments involving histology or immunohistochemistry, the figures shown are representative of at least three experiments (histologic or immunohistochemistry coloration) performed on different experimental days on the tissue sections collected from all the animals in each group. Data sets were examined with one- or two-way analysis of variance, and individual group means were then compared with Student unpaired *t* test. For the arthritis studies, Mann–Whitney *U* test (two-tailed, independent) was used to compare medians of the arthritic indices [22]. A *P* value of less than 0.05 was considered significant.

## Results

### Effect of PEA-LUT formulation therapy in the development of CIA

**Figure 1:**
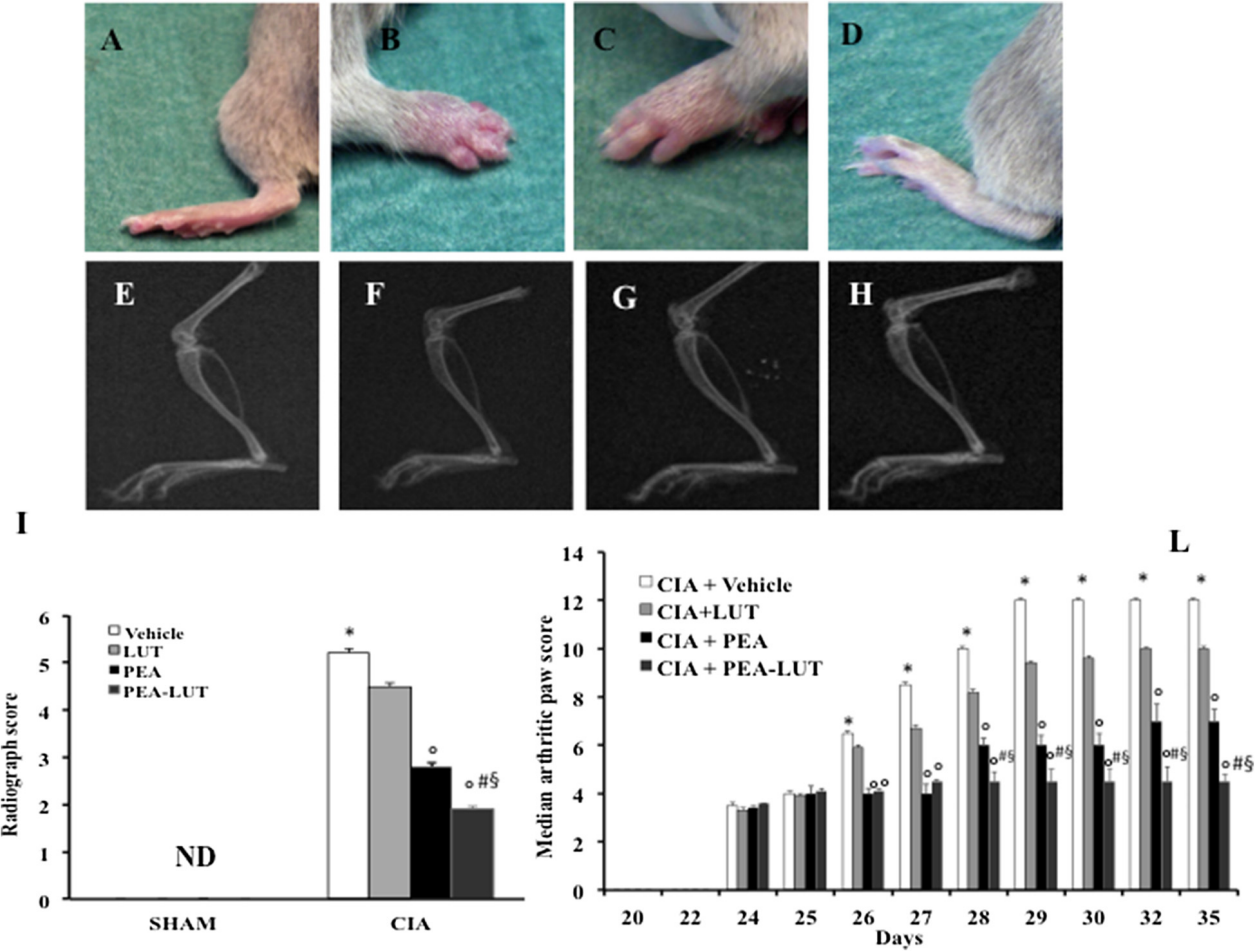
**Effect of PEA-LUT combination therapy on the clinical expression of CIA and on radiographic analysis.** No clinical signs were observed in sham mice **(A)**. CIA developed rapidly in mice immunized with CII and clinical signs like periarticular erythema and edema **(B)**. Hindpaw erythema and swelling increased in frequency and severity in a time-dependent mode **(L)**. CIA-PEA-treated mice demonstrated a significant reduction in the clinical signs of CIA **(C)**. Co-ultramicronized PEA + LUT formulation showed an enhanced reduction of clinical signs of CIA **(D)**. In addition, radiographic analysis was evaluated. No evidence of pathology in the femoral growth plate or in the tibiotarsal joints of normal mice **(E, I)**. Hindpaws from CII-immunized (35 days) vehicle-treated mice showed bone resorption in the femoral growth plate as well as in the tibiotarsal joints **(F, I)**. PEA-treated mice showed less bone erosion in the femoral growth plate, as well as in the tibiotarsal joints of CIA mice **(G, I)**. A significant difference was showed between PEA and PEA-LUT combination therapy as well as between PEA-LUT combination therapy and LUT-alone treatment **(H, I)**. Figure is representative of at least three experiments performed on different days. Values are expressed as mean ± SEM of 20 animals for each group. *P < 0.01 versus sham-control. °*P* < 0.01 versus CIA. ^*#*^*P* < 0.01 versus CIA-PEA. ^*§*^*P* < 0.01 versus CIA-LUT.

**Figure 2:**
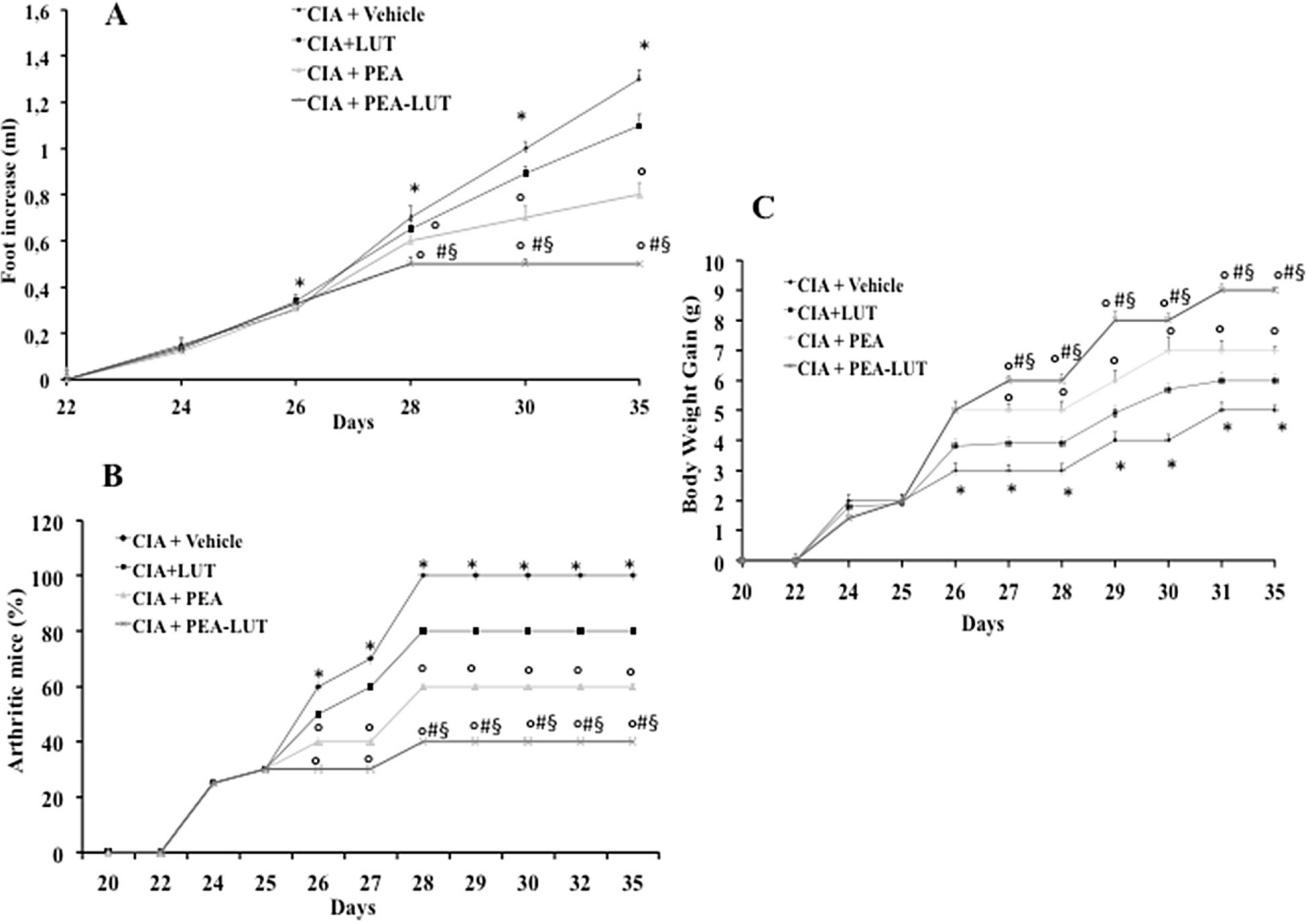
**Effect of PEA- LUT combination therapy on paw edema and body weight.** CIA developed rapidly in mice immunized with CII, leading to a 100% incidence of CIA at day 28 **(B)**. Swelling of hindpaws **(A)** over time was measured at 2-day intervals. Beginning on day 25, the CII-challenged mice gained significantly less weight, and this trend continued through day 35 **(C)**. CIA**-**PEA mice demonstrated a significant reduced incidence of weight loss **(C),** as well as less paw edema (A). CIA**-**LUT mice did not demonstrate a reduced incidence of weight loss **(C)** as well as less paw edema (A) compared with the PEA group. Furthermore, the combination therapy with PEA and LUT enhanced the reduction of incidence of body-weight loss and paw edema **(A, C)**. Figure is representative of all the animals in each group. Values are expressed as mean ± SEM of 20 animals for each group. *P < 0.01 versus Sham-control. °*P* < 0.01 versus CIA. ^*#*^*P* < 0.01 versus CIA-PEA. ^*§*^*P* < 0.01 versus CIA-LUT.

To imitate the clinical scenario of RA, mice were subjected to CIA. CIA developed rapidly in mice immunized with CII, and clinical signs (periarticular erythema and edema) (Figure 1B) of the disease first appeared in hindpaws between 24 and 26 days after challenge, leading to a 100% incidence of CIA at day 28 (Figure 2B). Hindpaw erythema and swelling increased in frequency and severity in a time-dependent mode, with maximum arthritis indices of approximately 12 observed between days 29 and 35 after immunization (Figure 1L) in CIA-control mice. The therapy with PEA-LUT significantly reduced the development of the inflammatory process (Figure 1D and L). A significant difference was found between the treatment with PEA-LUT and the higher dose of PEA alone (10 mg/kg) (Figure 1C and L), as well as between the PEA-LUT and LUT-alone treatment (1 mg/kg) (data not shown). Neither the clinical signs nor histopathologic features of CIA were observed in the paws of sham controls during the evaluation period (Figure 1A). PEA LUT significantly reduced the arthritis index (Figure 2B). A significant difference was found between the PEA-LUT therapy and the higher dose of PEA alone (10 mg/kg) (Figure 2B). No significant protection was found in the animal subjected to CIA treated with LUT (1 mg/kg, Figure 2B).

The data in Figure 2A demonstrate a time-dependent increase in hindpaw (each value represents the mean values of both hindpaws) volume (in milliliters) in mice immunized with CII. Maximum paw volume occurred by day 35 in the CII-immunized mice. Treatment with PEA-LUT exhibited a continuously significant (*P* < 0.01) suppression of hindpaw swelling from day 26 to 35 after immunization, achieving a maximal response of 75% from days 28 to 35 (Figure 2A). A significant difference was found between the higher dose of PEA alone (10 mg/kg) and the combination therapy (Figure 2A), as well as between the PEA-LUT and LUT-alone treatment (1 mg/kg) (Figure 2A). No significant inhibition of the paw-edema formation was found in the animals subjected to CIA treated with LUT (1 mg/kg, Figure 2A). No increases in hindpaw volume over time were observed with normal control (data not shown).

The rate and the absolute gain in body weight were comparable in sham-control and in CIA-control mice in the first week (data not shown). From day 25, the CII-challenged mice gained significantly less weight than the sham-control mice, and this trend continued through day 35 (Figure 2C). PEA-LUT treatment determined a significant increase of the body weight compared with the vehicle treatment in CIA-control mice (Figure 2C). A significant difference was found between the higher dose of PEA alone (10 mg/kg) and the combination therapy (Figure 2C), as well as between the PEA-LUT and LUT-alone treatment (1 mg/kg, Figure 2C). The treatment with LUT (1 mg/kg) did not exert any significant effect on the body-weight gain in the animals subjected to CIA (Figure 2C).

### PEA-LUT formulation therapy increases motor activity in CIA

**Figure 3:**
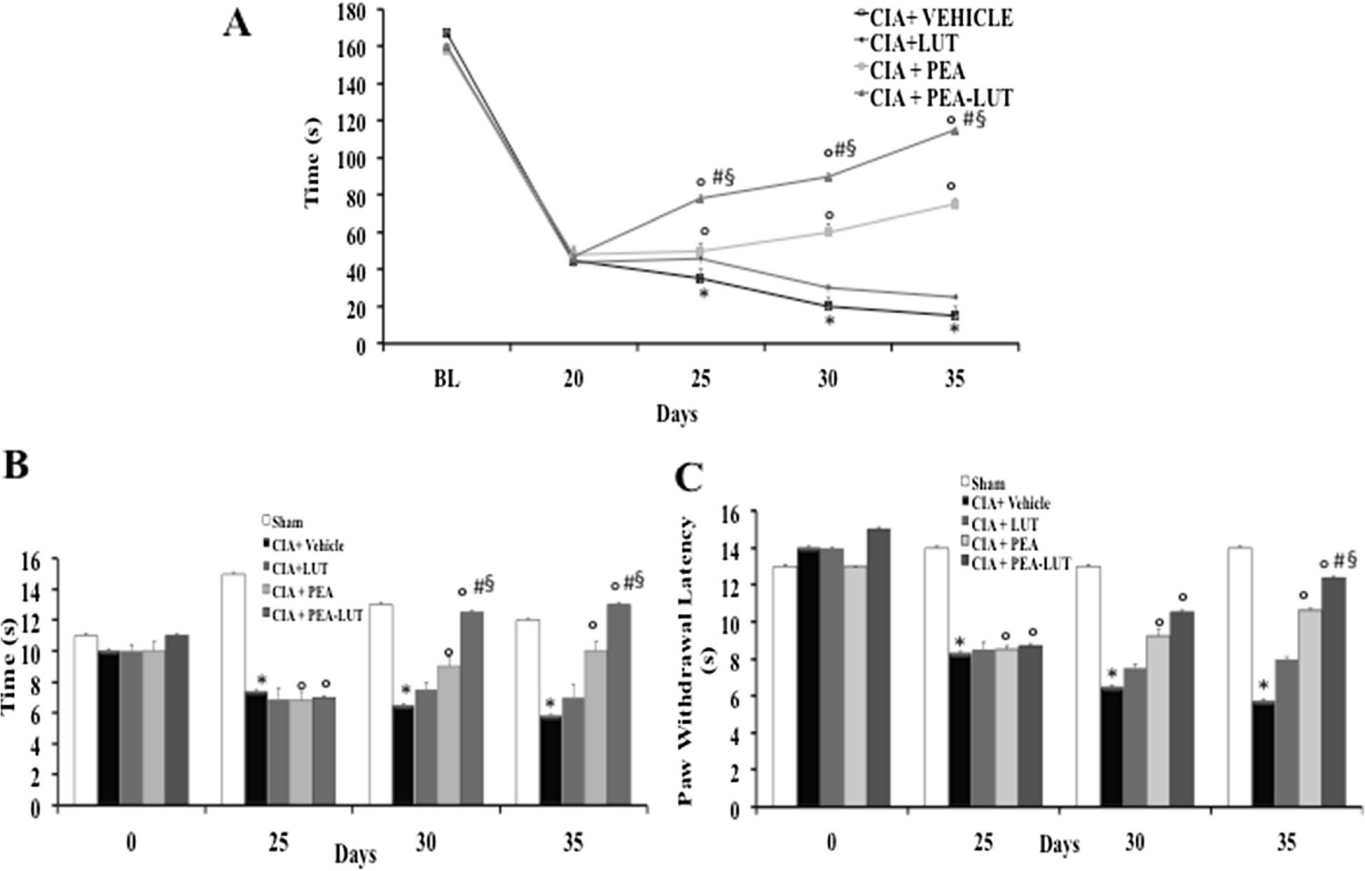
**Effect of PEA- LUT combination therapy on locomotor activity and pain evaluation.** CIA-subjected mice shortened the time to stay on a rotating rod compared with sham mice **(A)**. Locomotor abilities on the rotarod are better maintained in CIA + PEA than in CIA + Vehicle mice **(A)**. Locomotor abilities on the rotarod are not better maintained in CIA + LUT than on CIA + PEA mice **(A)**. A significant difference in locomotor activity was found between PEA and PEA-LUT combination therapy, as well as between PEA LUT combination therapy and LUT-alone treatment **(A)**. In addition, pain evaluation in CIA + vehicle, CIA-LUT, CIA + PEA, CIA + PEA-LUT, and sham mice was measured by a hotplate test and a plantar test. Measurements were recorded in mice able to ambulate. CIA + vehicle mice exhibit increased pain sensitivity and thermal hyperalgesia compared with normal controls. **(B, C)**. PEA treatment reduced significantly pain sensitivity and thermal hyperalgesia in CIA-PEA-treated mice **(B, C)**. LUT treatment did not significantly reduce pain sensitivity and thermal hyperalgesia compared with the PEA group **(B, C)**. The combination therapy with PEA-LUT enhanced the reduction of pain sensitivity and thermal hyperalgesia compared with a higher dose of PEA **(B, C)**. Figure is representative of all the animals in each group. Values are given as mean ± SEM of 20 animals for each group. *P < 0.01 versus Sham-control. °*P* < 0.01 versus CIA. ^*#*^*P* < 0.01 versus CIA-PEA. ^*§*^*P* < 0.01 versus CIA-LUT.

In a previous study, we demonstrated that PEA-LUT treatment is able to alleviate many of the clinical and neuropathologic features of depression [32], but to our knowledge, PEA-LUT has not been investigated in this regard in CIA. Therefore, we next assessed how prolonged daily treatment with PEA-LUT affected the gross locomotor ability, assessed by performance on a nonaccelerating rotarod, in CIA mice. As shown in Figure 3A, PEA-LUT treatment significantly reduced the motor impairment in CIA mice. Moreover, a significant difference on the motor-function impairment was found between the PEA-LUT therapy and the higher dose of PEA alone (10 mg/kg) and between PEA-LUT therapy and LUT at a dose of 1 mg/kg (Figure 3A). Treatment with LUT (1 mg/kg) did not significantly ameliorate the locomotor ability in the animals subjected to CIA (Figure 3A).

### Effect of PEA-LUT formulation therapy on pain sensitivity and thermal hyperalgesia in CIA

In the next step, the effect of PEA-LUT therapy on pain sensitivity was tested by subjecting mice to hotplate testing and recording latency to a response (Figure 3B). At day 25 after CIA induction, CIA + vehicle mice exhibited increased pain sensitivity compared with normal controls (Figure 3B). Moreover, between days 30 and 35 after immunization, PEA-LUT-treated mice with CIA had response times that were comparable to normals (Figure 3B). Furthermore, at day 25 after CIA induction, mice became hypersensitive to noxious heat (thermal hyperalgesia), as evidenced by a significant reduction in hindpaw withdrawal latency, with a maximum hypersensitive response observed between days 30 and 35 after immunization in CIA-control mice (Figure 3C). This CIA-induced hyperalgesia was reduced by daily treatment with PEA-LUT (Figure 3C). Moreover, a significant difference on the pain-sensitivity impairment was found between the PEA-LUT therapy and the higher dose of PEA alone (10 mg/kg) (Figure 3B, C), as well as between the PEA-LUT and LUT-alone treatment (1 mg/kg, Figure 3B, C).

### Effect of PEA-LUT formulation therapy on histopathology and radiographic analysis of CIA

**Figure 4:**
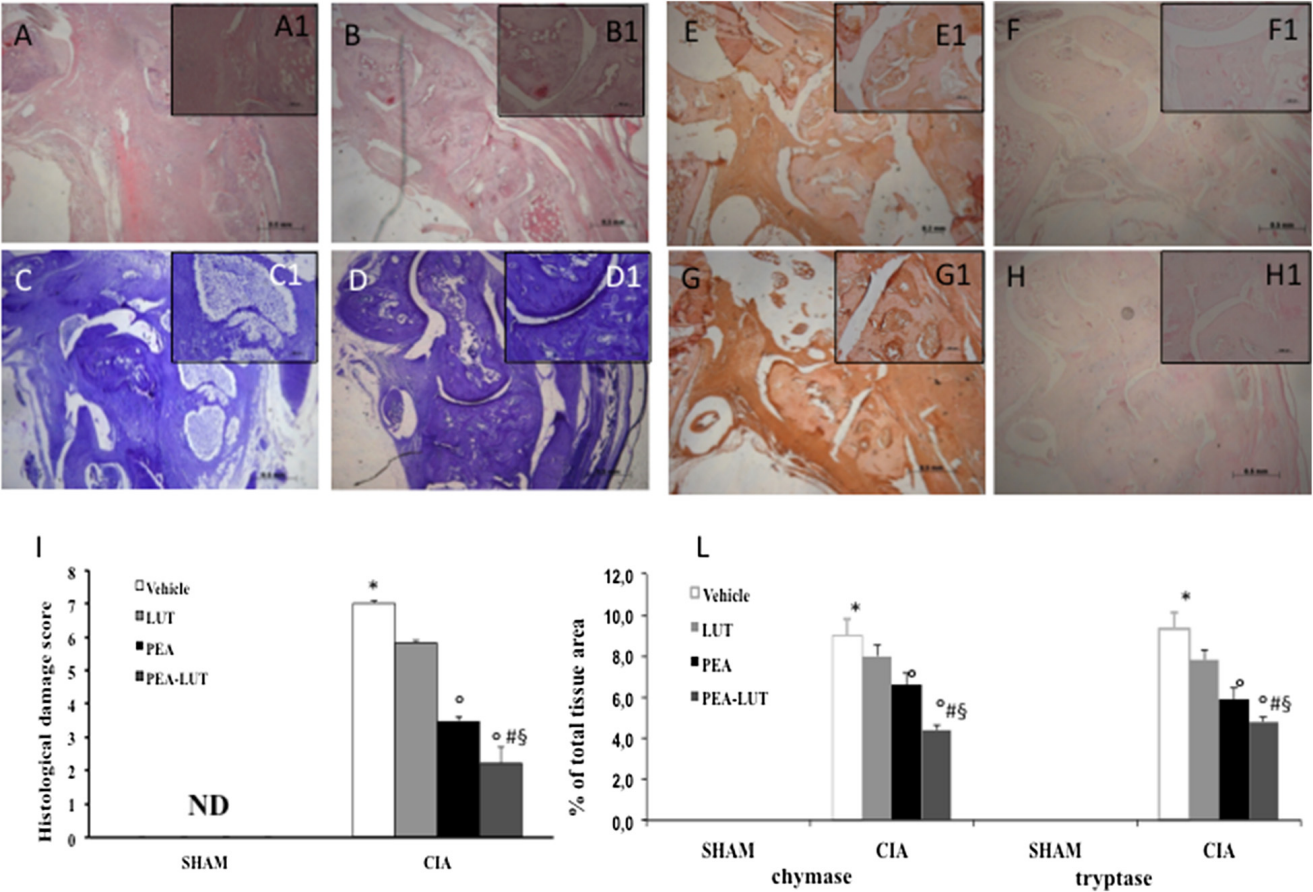
**Morphologic changes of CIA.** Representative hematoxylin/eosin-stained section of the joint was examined with light microscopy. The histologic evaluation of a joint from CIA-control mice **(A, A1,** and **I)** revealed inflammatory cell infiltration and bone erosion. The histologic alterations were significantly reduced in the tissues from CIA-PEA-LUT treated mice **(B, B1,** and **I)**. Toluidine blue staining was also performed. A significant mast cell infiltration was observed in joint tissues of CIA-subjected mice **(C, C1)** compared with sham animals (data not shown). PEA-LUT enhanced the reduction of mast cell infiltration **(D, D1)**. In addition, a significant increase in chymase and tryptase expression was found mainly in the joint tissues collected after CIA induction **(E, E1, G, G1,** and **L)**. Chymase and tryptase expression was significantly attenuated in the joint from CIA-PEA-LUT-treated mice **(F, F1, H, H1,** and see **L)**. Densitometry analysis of immunocytochemistry photographs (*n* = 5) for chymase and tryptase from paw sections was assessed **(L)**. Data are expressed as percentage of total tissue area. *P < 0.01 versus Sham-control. °*P* < 0.01 versus CIA. ^*#*^*P* < 0.01 versus CIA-PEA. ^*§*^*P* < 0.01 versus CIA-LUT.

The histologic evaluation (at day 35) of the paws from vehicle-treated mice revealed signs of severe arthritis, with bone erosion (Figure 4A, A1, and see histologic score I). In addition, severe or moderate necrosis was observed (Figure 4A, A1, and see histologic score I). The bone erosion and the necrosis were significantly reduced in the joint from PEA-LUT-treated mice (Figure 4B, B1, see for histologic score I). A significant difference was found between the higher dose of PEA alone (10 mg/kg) and the combination therapy, as well as between the PEA-LUT and LUT-alone treatment (1 mg/kg, data not shown). Treatment with LUT (1 mg/kg) did not reduce the histologic alteration in the animals subjected to CIA (data not shown). No histologic damage was found in sham animals (data not shown).

A radiographic examination of knee joint and femoral growth plate in the femur from vehicle-treated mice at 35 days after CII immunization revealed bone erosion (Figure 1F; see radiograph score I). Significantly less bone resorption was observed in the PEA-treated mice (Figure 1G, and see radiograph score I). Treatment with LUT (1 mg/kg) did not reduce significantly the bone resorption in the animals subjected to CIA (data not shown). A significant difference was found between the higher dose of PEA alone (10 mg/kg) and the combination therapy (Figure 1H, and see radiograph score I), as well as between the PEA-LUT and LUT-alone treatment (1 mg/kg, data not shown). No evidence of pathology was found in sham mice (Figure 1E, and see radiograph score I).

### Effect of PEA-LUT formulation therapy on mast cells degranulation during CIA

The better to study the mast cell infiltration during CIA, the joint tissues were stained with toluidine blue. In particular, a significant presence of mast cells was observed in the joint tissues collected at day 35 after CIA induction (Figure 4C), mainly localized in the articular space (see particles 4 C1). On the contrary, significantly less mast cell infiltration was observed in the joint tissues from CIA-subjected mice that were been treated with PEA-LUT (Figure 4D, D1). A significant difference was found between the higher dose of PEA alone (10 mg/kg) and the combination therapy, as well as between the PEA-LUT and LUT-alone treatment (1 mg/kg, data not shown). No resident mast cells were found in the joint tissues from sham-treated mice (data not shown).

Moreover, to test whether PEA-LUT treatment may modulate and direct the inflammatory response through the regulation of the serine peptidases, we analyzed with immunohistochemistry the joint expression of chymase and tryptase. No staining for chymase and tryptase occurred in the joint tissues obtained from the sham-treated mice (data not shown). A substantial increase in chymase and tryptase expression was found mainly localized in mast cells in the joint tissues collected at 35 days after CIA induction (Figure 4E, E1, G, G1, and see L). Joint expression of chymase and tryptase was significantly attenuated in the joints from CIA mice that received PEA-LUT treatment (Figure 4F, F1, H, H1, and see L). A significant difference was found between the higher dose of PEA alone (10 mg/kg) and the combination therapy, as well as between the PEA-LUT and LUT-alone treatment (1 mg/kg, data not shown).

### Effect of PEA-LUT formulation therapy on cytokines, chemokines expression, and neutrophil infiltration

**Figure 5:**
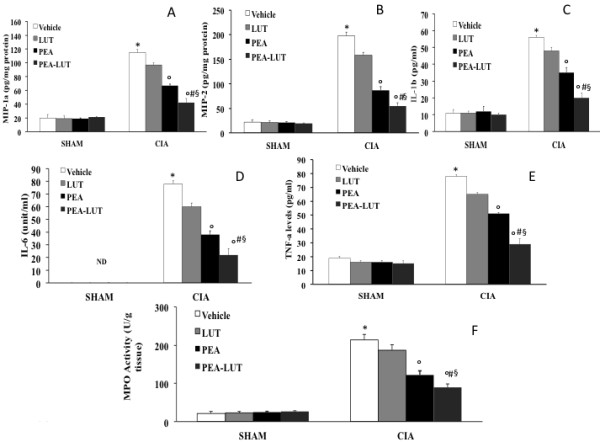
**Effect of PEA-LUT combination therapy on cytokines, chemokine expression, and neutrophil infiltration.** A substantial increase in the expression of MIP-1α **(A)**, MIP-2 **(B)**, IL-1β **(C)**, IL-6 **(D)**, TNF-α **(E)**, and MPO activity **(F)** was found in CIA-control mice 35 days after CII immunization. CIA-PEA-treated mice demonstrated a significant reduction in the expression of MIP-1α **(A)**, MIP-2 **(B)**, IL-1β **(C)**, IL-6 **(D)**, TNF-α **(E)**, and MPO activity **(F)**. CIA-LUT-treated mice did not significantly reduce the expression of MIP-1α **(A)**, MIP-2 **(B)**, IL-1β **(C)**, IL-6 **(D)**, TNF-α **(E)**, and MPO activity **(F)**. The combination therapy with PEA-LUT significantly reduced the expression of MIP-1α **(A)**, MIP-2 **(B)**, IL-1β **(C)**, IL-6 **(D)**, TNF-α **(E)**, and MPO activity **(F)**. Values are shown as mean ± SEM of 20 animals for each group. *P < 0.01 versus Sham-control. °*P* < 0.01 versus CIA-control. ^*#*^*P* < 0.01 versus CIA-PEA. ^*§*^*P* < 0.01 versus CIA-LUT.

We initiated studies to assess the effect of PEA-LUT therapy on the expression of chemokines into the inflamed joints during the development of CIA. As shown in Figure 5A, B, the expression of MIP-lα and MIP-2, measured with ELISA, was significantly increased in the joint 35 days after CII immunization. MIP-1α and MIP-2 levels in CIA mice that were treated with PEA-LUT on day 35 were significantly reduced in comparison with vehicle-treated CIA mice (Figure 5A, B). Assessment of neutrophil infiltration into the inflamed joint tissue was also performed by measuring MPO activity. It was significantly elevated 35 days after CII immunization in vehicle-treated CIA mice (Figure 5F), whereas in the CIA mice treated with PEA-LUT therapy, MPO activity was markedly reduced (Figure 5F). A significant difference was found between the higher dose of PEA alone (10 mg/kg) and the combination therapy, as well as between the PEA-LUT and LUT-alone treatment (1 mg/kg, Figure 5A, B, F).

To test whether PEA-LUT modulates the inflammatory process through the regulation of cytokine secretion, we analyzed the plasma levels of the proinflammatory cytokines TNF-α, IL-1β, and IL-6. A substantial increase in TNF-α, IL-1β, and IL-6 (Figure 5C, D, E) production was found in CIA-control mice 35 days after CII immunization. Levels of TNF-α, IL-1β, and IL-6 (Figure 5C, D, E) were significantly reduced in CIA mice treated with PEA-LUT therapy. No significant difference was found between the higher dose of PEA alone (10 mg/kg) and the combination therapy, as well as between the PEA-LUT and LUT-alone treatment (1 mg/kg, Figure 5C, D, E).

### Effect of PEA-LUT formulation therapy on nitrotyrosine formation and lipid peroxidation

**Figure 6:**
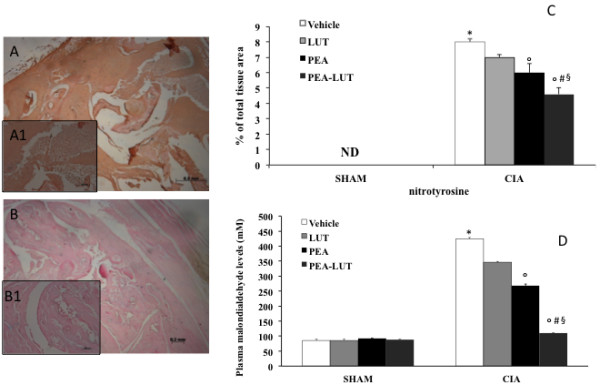
**Effect of PEA-LUT combination therapy on nitrotyrosine immunostaining and MDA levels.** A marked increase in nitrotyrosine (**A**, see particularly **A1** and **C**), staining was evident in the paw 35 days after initiation of CIA. A marked reduction was seen in the immunostaining for nitrotyrosine (**B**, see particularly **B1** and **C**) in the paws of CIA-PEA-LUT mice. Densitometry analysis of immunocytochemistry photographs (*n* = 5) for nitrotyrosine from paw sections was assessed **(C)**. The assay was carried out by using Optilab Graftek software on a Macintosh personal computer (CPU G3-266). Data are expressed as percentage of total tissue area. *P < 0.01 versus Sham-control. °*P* < 0.01 versus CIA. In addition, MDA levels, a marker of lipid peroxidation, were evaluated. A substantial increase in MDA levels **(D)** was found in CIA-control mice 35 days after CII immunization. CIA-PEA-treated mice demonstrated a significant reduction in MDA levels **(D)**. The combination therapy with PEA-LUT enhanced the reduction in MDA levels **(D)**. Values are given as mean ± SEM of 20 animals for each group. *P < 0.01 versus Sham-control. °*P* < 0.01 versus CIA-control. ^*#*^*P* < 0.01 versus CIA-PEA. ^*§*^*P* < 0.01 versus CIA-LUT.

The release of free radicals and oxidant molecules during chronic inflammation has been suggested to contribute significantly to the tissue injury [33]. On day 35, a positive staining for nitrotyrosine, a marker of nitrosative injury, was found in the joints of vehicle-treated CIA-control mice (Figure 6A, A1, and see C). The therapy with PEA-LUT significantly reduced the formation of nitrotyrosine (Figure 6B, B1, and see C). A significant difference was found between the higher dose of PEA alone (10 mg/kg) and the combination therapy, as well as between the PEA-LUT and LUT-alone treatment (1 mg/kg, data not shown).

In addition, at 35 days after CIA induction, thiobarbituric acid-reactant substance levels were measured in the plasma as an indicator of lipid peroxidation. A significant increase of thiobarbituric acid-reactant substances (Figure 6D) was observed in the plasma collected at 35 days after CIA induction from mice subjected to CIA when compared with sham-operated mice. Thiobarbituric acid-reactant substances (Figure 6D) were significantly attenuated in CIA-mice treated with PEA-LUT. Please note that a significant difference was found between the higher dose of PEA alone (10 mg/kg) and the combination therapy (Figure 6D), as well as between the PEA-LUT and LUT-alone treatment (1 mg/kg, Figure 6D).

## Discussion

ROSs have a crucial role in the pathogenesis of inflammatory diseases such as rheumatoid arthritis [34,35]. The flavonoid, apigenin, which has a structure similar to that of luteolin, showed an antidepressant-like effect [36] and a protective effect against endoplasmic stress-induced neuronal cell death [36]. These findings suggest the possibility that luteolin may be protective against oxidative stress-induced inflammation and cell damage. With this aim in mind, we applied the endocannabinoid congener PEA and the flavonoid luteolin to counteract the inflammatory process associated with RA. Our results demonstrated that co-ultramicronized PEA + luteolin formulation (PEA-LUT) is protective in a mouse model of collagen-induced arthritis. The protective effects of PEA-LUT were not limited to an overall antiinflammatory effect, but included significant protection of cartilage/bone compared with that in untreated collagen-immunized animals, as well as inhibition of proinflammatory cytokines known to be involved in the human disease. Through both histologic and radiographic evaluations, we found that PEA-LUT was significantly protective on the cartilage and bone in tibiotarsal joints of mice immunized with CII.

PEA has been shown to be effective in several experimental models of inflammation, of both immunogenic and neurogenic origin [37,38]. We recently demonstrated that PEA treatment significantly reduced spinal cord injury in mice [39]. Despite its various pharmacologic properties, the cellular/receptor mechanism responsible for the actions of PEA is still debated. Mazzari *et al*. [37] demonstrated that *in vivo* antiinflammatory effects of PEA were due to downregulation of mast cell (MC) degranulation. The ability of MCs to respond to a wide range of infectious and chemical stimuli facilitates their key functions in immunity and the response to tissue injury, by promoting a rapid release of proinflammatory mediators, mediators of hyperalgesia, and itch mediators [40]. MCs were divided into two subtypes, depending on the variable content of the neutral serine proteases, tryptase and chymase. We report here that CIA caused a significant infiltration and activation of MCs in the joint at 35 days after induction, whereas treatment with PEA-LUT significantly reduced both the infiltration and the activation. These observations are in agreement with other studies, which have shown that PEA is an effective tool to control mast cell hyperactivity, which occurs in inflammation, inflammatory hyperalgesia [41], neuropathic hyperalgesia [42], and spinal and brain trauma [19,26]. It has been demonstrated that several cytokines also appear to direct cell-to-cell communication in a cascade fashion during CIA, such as: IL-1 [43], TNF-α [44], and IL-6 [45]. TNF-α and IL-1β are initiators of the nuclear factor (NF-κB) activation cascade [34] and are under its transcriptional control, constituting a positive-feedback loop. Recent studies observed that the luteolin or PEA decreased the activation of the NF-κB system in different experimental models [46,47].

We confirmed that the proinflammatory cytokines (IL-1β, IL-6, and TNF-α), as well as the chemokines (MIP-Iα and MIP-2), are expressed at sites of inflamed joints and likely contribute to the progression of chronic joint inflammation. It has been demonstrated that MCP-1, MIP- Iα, and MIP-Iβ are differentially chemotactic for lymphocyte subsets [2] and are expressed in tissue from the inflamed joints of patients with rheumatoid arthritis [48,49]. Interestingly, by using PEA-LUT, we demonstrated a more pronounced inhibition of the release of proinflammatory cytokines and chemokines and a reduction of leukocyte infiltration measured by MPO activity in comparison with PEA alone.

The role of ROSs and, in particular, of superoxide in degradation of cartilage and bone is well documented [11,50]. Cartilage is sensitive to degradation by superoxide, and SOD strongly inhibits this degradation; evidence exists to support a link between chondrocyte lipid peroxidation and cartilage oxidation/degradation [11]. In this report, an intense immunostaining of nitrotyrosine formation and a significant lipid peroxidation also suggested that a structural alteration of joint had occurred, most probably because of the formation of highly reactive nitrogen derivatives.

We demonstrated here that PEA-LUT reduced the nitrotyrosine and the lipid peroxidation formation during CIA. Several studies demonstrated that luteolin also exerts antioxidant properties [51]. This effect on nitrotyrosine formation and lipid peroxidation by PEA-LUT was significantly more pronounced in comparison with PEA alone.

In previous studies, we demonstrated that combination therapy by using a potent M40403 SODm and clinically used drugs (for example, dexamethasone or methotrexate) significantly exerts an important beneficial antiinflammatory effect by blocking the possible progression of an emerging arthritis with the reduction of a DMARS effective dose [11]. Similarly, in the present study, we demonstrated that PEA-LUT, when given at the onset of the disease, reduced paw swelling, the clinical score, and the histologic severity. Amelioration of joint disease was associated with inhibition of pain, which is a key player in RA. Thus, these potent analgesic and antiinflammatory effects observed with PEA-LUT were in contrast to those observed with the individual substances administered at lower doses (data not shown).

## Conclusions

RA is a complex chronic inflammatory disease dependent on multiple interacting environmental and genetic factors, making it difficult to understand its pathogenesis and thereby to find effective therapies. This new pharmacologic approach with PEA-LUT combination therapy may represent new and useful pharmacologic tools for the therapy for chronic inflammation.

## Abbreviations

CFA: Complete Freund adjuvant; CIA: Type II collagen-induced arthritis; CII: Collagen type II; IL-1β: Interleukin-1β; IL-6: Interleukin-6; LUT: Luteolin; MC: Mast cell; MPO: Myeloperoxidase; NF-κB: Nuclear factor-κB; PEA: *N*-palmitoylethanolamine; RA: Rheumatoid arthritis; ROS: Reactive oxygen species; TNF-α: Tumor necrosis factor-α.

## Competing interests

The authors declare that they have no competing interests.

## Authors’ contributions

DB and SC participated in research design and performed statistical analysis. AP and VM also participated in study design and coordination. DI and RD carried out immunoassay and histologic analysis and helped to draft the manuscript. AA, EE, and MC also conducted experiments and helped to draft the manuscript. DI, EE, and SC contributed to the writing of the manuscript. All authors read and approved the final manuscript.
