# Erratum to: Palmitoylethanolamide and luteolin ameliorate development of arthritis caused by injection of collagen type II in mice

**DOI:** 10.1186/s13075-016-0999-9

**Published:** 2016-05-05

**Authors:** Daniela Impellizzeri, Emanuela Esposito, Rosanna Di Paola, Akbar Ahmad, Michela Campolo, Angelo Peli, Valeria Maria Morittu, Domenico Britti, Salvatore Cuzzocrea

**Affiliations:** Department of Biological and Environmental Sciences, University of Messina, Messina, Italy; Clinical Veterinary Department Alma Mater Studiorum, University of Bologna, Bologna, Italy; Department of Health Sciences V. le Europa, Campus S. Venuta, Germaneto, Catanzaro, 88100 Italy; Manchester Biomedical Research Centre, Manchester Royal Infirmary, University of Manchester, Manchester, UK

The authors would like to issue an erratum for this article [1], and would like to declare the following competing interests which we inadvertently failed to include in our original publication. The authors would like to apologise for this omission.

In addition, a detailed method for co-ultramicronization process is reported:

*Co-ultramicronization process was performed in a jet-mill equipment endowed with a chamber of 300 mm in diameter which operates with “spiral technology” and was driven by compressed air at 10-12 bars. The crashing is determined by the high number of collisions that occurs among particles, as a result of the high level of kinetic – not mechanical - energy. This process was effective not only in reducing the products particle size, but also in modifying their crystalline structure. Observations by scanning electron microscopy (SEM) shows an intimate intermixing of the two components of the composite, while analysis throw differential scanning calorimetry (DSC) and X-ray diffraction (XRD) have documented the transformation in a new crystalline form different from the original two, definable with “a higher energy content form.” The composite shows the following particle size distribution: 96 % <10 μm; 80 % <5 μm; 40 % <2 μm (J Neuroinflammation 2013 10:91).*
